# Elevated lipoprotein(a) and adverse pregnancy outcomes

**DOI:** 10.1097/MS9.0000000000005411

**Published:** 2026-09-10

**Authors:** Ugochukwu Ebubechukwu, Toluwalase Awoyemi, Onyinye Ugoala, Ike Kendra Bolakale-Rufai, Valentine C. Nriagu, Chukwuemelie Okeke, Chukwuebuka Stanley Asogwa, Queen Ugochinyere Alu, Oluwaremilekun Tolu-Akinnawo, Emmanuel Daniel, Christabel Uche-Orji, Oluwaseun Adeleke, Anandita Agarwala, Lynn M. Yee

**Affiliations:** a Department of Internal Medicine, SUNY Downstate Health Sciences University, Brooklyn, NY, USA; b Department of Internal Medicine, Northwestern University Feinberg School of Medicine, Chicago, IL, USA; c Department of Internal Medicine, Texas Tech University Health Sciences Center, Amarillo, TX, USA; d Department of Medicine, Maimonides Medical Center, Brooklyn, NY, USA; e Department of Physiology, St George’s University School of Medicine, True Blue, Grenada; f College of Medicine, University of Ibadan, Ibadan, Nigeria; g Cardiology Division, Alex Ekwueme Federal University Teaching Hospital, Abakaliki, Nigeria; h Department of Internal Medicine, Meharry Medical College, Nashville, Tennessee, USA; i Department of Internal Medicine, Trinity Health Ann Arbor, Ann Arbor, Michigan, USA; j Department of Internal Medicine, Sumy State University, Sumy, Ukraine; k Center for Cardiovascular Disease Prevention, Baylor Scott and White Health Heart Hospital Baylor Plano, Plano, TX, USA; l Division of Maternal-Fetal Medicine, Department of Obstetrics and Gynecology, Northwestern University Feinberg School of Medicine, Chicago, IL, USA

**Keywords:** fetal growth restriction, gestational hypertension, lipoprotein(a), Lp(a), preeclampsia, pregnancy outcomes, preterm birth

## Abstract

Lipoprotein(a) [Lp(a)] is gaining attention as a potential marker for adverse pregnancy outcomes, in addition to its well-established role in cardiovascular disease. This review explores the evolving understanding of Lp(a) in pregnancy, focusing on its links to hypertensive disorders of pregnancy, gestational diabetes mellitus, fetal growth restriction, and preterm birth, while also addressing diagnostic and management challenges. During pregnancy, Lp(a) levels rise substantially, nearly doubling between weeks 10 and 35. Although this increase is universal, its clinical implications are unclear. Lp(a) exerts pro-inflammatory, pro-thrombotic, and pro-atherogenic effects, the mechanisms of which are discussed in detail in the body of this review. However, research into Lp(a)’s specific role in increasing the risk of adverse pregnancy outcomes, such as hypertensive disorders of pregnancy, has produced mixed results, highlighting the need for further investigation. Routine screening for Lp(a) during pregnancy is not standard practice, though some experts suggest that it could be beneficial, particularly for high-risk pregnant people. Similar to managing other cardiovascular disease risk factors, managing elevated Lp(a) during pregnancy requires a comprehensive approach, from preconception counseling to lifestyle adjustments such as improving diet, increasing physical activity, and quitting smoking. Pharmacological options like statins and proprotein convertase subtilisin/kexin type 9 inhibitors have shown promise in non-pregnant populations, but their safety during pregnancy remains a concern, limiting their use. Further research is needed to clarify Lp(a)’s role in adverse pregnancy outcomes and to identify safe, effective treatments for managing elevated Lp(a) levels before and during pregnancy.

## Introduction

Elevated lipoprotein(a) [Lp(a)] is a well-established risk factor for atherosclerotic cardiovascular disease (ASCVD) in the general population, with recent guidelines advocating for its routine screening^[^1–3^]^. Lp(a) consists of low-density lipoprotein (LDL) bound to apolipoprotein(a) via apolipoprotein B100, and levels generally stabilize by age five, remaining constant except in specific conditions, including inflammation, pregnancy, or kidney disease^[^2,4,5^]^. Extensive data have also shown that Lp(a) is involved in thrombotic and atherosclerotic processes linked to increased cardiovascular disease risk^[^6,7^]^. Sex differences in Lp(a)-related risks are notable, with females facing a higher risk of ASCVD than males at similar Lp(a) levels^[^2,8,9^]^. During pregnancy, Lp(a) levels rise substantially, returning to baseline within 6 months postpartum^[^1,2,9,10^]^. These increases during pregnancy are thought to be influenced by hormonal shifts, particularly the rise in estrogen, which may affect Lp(a) synthesis and clearance[11]. Initially thought to aid only in hemostasis and wound healing, Lp(a) is now understood to induce both placental and systemic endothelial dysfunction, with apo(a)’s structural similarity to plasminogen promoting a prothrombotic state^[^1,3^]^.

HIGHLIGHTS

- Lipoprotein(a) [Lp(a)] is emerging as a potential marker for adverse pregnancy outcomes, including hypertensive disorders of pregnancy, gestational diabetes, fetal growth restriction, and preterm birth.
- Lp(a) levels nearly double between weeks 10 and 35 of pregnancy, but the clinical significance of this increase remains unclear.
- Pro-inflammatory, pro-thrombotic, and pro-atherogenic properties of Lp(a) may contribute to vascular and placental dysfunction, although research findings are mixed.
- Routine screening for Lp(a) in pregnancy is not standard practice, and pharmacological management is limited due to safety concerns, highlighting the need for further research.

Despite these insights, research linking serum Lp(a) levels to adverse pregnancy outcomes such as preeclampsia (PE), gestational hypertension, and fetal growth restriction (FGR) remains inconsistent[2]. Inconsistent findings and a paucity of data have limited the creation of clear guidelines for managing Lp(a) in people with high-risk pregnancies. This review evaluates current evidence on Lp(a) and adverse pregnancy outcomes, explores the mechanisms of Lp(a)-mediated vascular dysfunction, and discusses diagnostic challenges, potential interventions, and future research directions. This review is compliant with the Transparency in the Reporting of Artificial Intelligence (TITAN) guidelines[12]. This narrative review adds to the existing literature by synthesizing the most recent evidence on Lp(a) in pregnancy, including updated guideline positions published since 2023, the mechanistic interplay between Lp(a) and maternal obesity, a gap not addressed in prior reviews, and the emerging therapeutic landscape relevant to future pregnancy-specific research.

## Literature search strategy

A narrative search of PubMed/MEDLINE, EMBASE, and the Cochrane Library was conducted from January 1990 to February 2025. Search terms applied individually and in combination using Boolean operators (AND, OR) included: “lipoprotein(a),” “Lp(a),” “lipoprotein a,” “apo(a),” “pregnancy,” “adverse pregnancy outcomes,” “preeclampsia,” “gestational hypertension,” “hypertensive disorders of pregnancy,” “fetal growth restriction,” “intrauterine growth restriction,” “preterm birth,” “gestational diabetes mellitus,” “placental dysfunction,” “cardiovascular risk,” and “maternal cardiovascular disease.” Eligible study designs included original research (cohort, case-control, cross-sectional, and randomized controlled trials), systematic reviews, meta-analyses, narrative reviews, and clinical guidelines, and were restricted to English-language publications. Case reports were included only where they provided mechanistic or clinical insight not available in primary research. The initial search identified approximately 420 records; after removing approximately 110 duplicates, approximately 310 titles and abstracts were screened. Approximately 111 full-text articles were assessed for eligibility; 60 met the inclusion criteria from the database search. A further 13 records were identified through manual searching of reference lists, bringing the total to 73 included sources, consistent with the reference list of this review. As this is a narrative rather than a systematic review, a formal PRISMA flow diagram was not produced; no formal risk-of-bias assessment was performed; and no quantitative meta-analysis was undertaken. The search strategy is reported in full to ensure methodological transparency, and the screening numbers provided are approximate, intended to characterize the scope of the literature search rather than to imply systematic rigor.

## Pathophysiological mechanisms

### Mechanisms of elevated Lp(a) in pregnancy

During pregnancy, Lp(a) levels rise as part of a broader shift in lipid metabolism, which also includes increases in total cholesterol, LDL cholesterol, and triglycerides to support fetal growth and development[13]. This elevation may be influenced by the insulin resistance that develops throughout gestation, thereby disrupting lipid metabolism and promoting the production of lipoproteins such as Lp(a)^[^13,14^]^. Heightened estrogen levels during pregnancy can reduce lipoprotein lipase activity, impairing Lp(a) clearance[13].

### Role of Lp(a) in vascular dysfunction and cardiovascular disease

The vascular effects of Lp(a) operate through several interconnected mechanisms: Lp(a) enhances the expression of vascular cell adhesion molecules and E-selectins, which facilitate the activation and transmigration of peripheral monocytes across the endothelium, contributing to foam cell formation^[^15,16^]^. Elevated Lp(a) levels are associated with impaired endothelium-dependent vasodilation, although direct causation remains unclear[17]. This vasodilation is mediated by nitric oxide (NO), which plays a protective role by inhibiting vascular smooth muscle cell proliferation and minimizing leukocyte adhesion[18]. Lp(a) disrupts NO production and action, exacerbating its atherogenic effects^[^19,20^]^. Additionally, Lp(a) exhibits pro-thrombotic properties by inhibiting the fibrinolytic process; its structural similarity to plasminogen allows it to compete for binding sites on fibrin, obstructing fibrinolysis and promoting thrombosis[21]. Lp(a) further inhibits t-PA-mediated plasminogen activation through direct competition^[^16,22^]^.

The interplay of these pro-atherogenic and pro-thrombotic effects is evident in clinical observations, such as those by Kamstrup *et al*, who found a stepwise increase in myocardial infarction risk associated with higher Lp(a) levels, predicting a three- to four-fold increase in risk among the general population[23]. Ramharack *et al* highlighted Lp(a)’s role in inflammatory states, showing that interleukin-6 (IL-6) enhances Lp(a) expression, while TGF-β1 and TNF-α inhibit it[24]. Lp(a) is studied as an acute-phase reactant because its synthesis is stimulated during inflammatory responses, which can lead to arterial wall inflammation and endothelial dysfunction[25]. Elevated Lp(a) levels have been linked to increased cardiovascular disease risk in patients hospitalized with COVID-19, and persistently high Lp(a) concentrations are observed in chronic inflammatory conditions^[^25,26^]^. Furthermore, Lp(a) levels tend to decrease with the use of anti-inflammatory therapies, such as methotrexate, highlighting its pro-inflammatory role^[^25,27^]^.

## Lp(a) and adverse pregnancy outcomes

### Association between Lp(a) and hypertensive disorders of pregnancy

The relationship between Lp(a) and PE has become a significant focus in research, especially regarding lipid metabolism’s role in this adverse pregnancy outcome. Some studies indicate elevated Lp(a) levels in people with PE compared to those without PE. For instance, a prospective observational study suggested that serum Lp(a) levels above 40.5 mg/dL in pregnant individuals with PE without severe features could predict progression to PE with severe features[28]. However, other research challenges this link; one study found no significant differences in Lp(a) levels between 91 people with PE and 40 healthy pregnant controls, leading the authors to conclude that high serum Lp(a) levels are unlikely to be a risk factor for PE[29]. A longitudinal investigation reported a significant increase in Lp(a) levels during normal pregnancy, with no notable differences between preeclamptic and healthy people at 32 weeks of gestation[30]. The discordance across studies likely reflects important methodological heterogeneity: studies vary considerably in sample size, gestational timing of Lp(a) measurement, outcome definitions, and whether confounders such as body mass index (BMI) and pre-existing renal disease were accounted for. Furthermore, most studies are observational and cannot establish causality. On balance, current evidence is insufficient to conclude that elevated Lp(a) is an independent risk factor for hypertensive disorders of pregnancy (HDP), and this uncertainty should be reflected in any clinical interpretation[31].

Furthermore, Lp(a) acts as an inflammatory lipoprotein, potentially inducing endothelial dysfunction in the systemic and placental vasculature[1]. This dysfunction could compromise placental arterial integrity, contributing to maternal hypertension and its maternal-fetal sequelae[30]. Additionally, genetic studies indicate that elevated Lp(a) levels may be associated with endothelial dysfunction, suggesting that inherited factors could predispose certain women to both high Lp(a) levels and an increased risk of PE[1]. Overall, the relationship between Lp(a) and PE remains uncertain, with conflicting evidence supporting both a potential link and no significant association. Further research is necessary to clarify the role of Lp(a) in PE and its potential implications for predictive and therapeutic strategies (Table 1; Figure 1)

**Figure 1.**
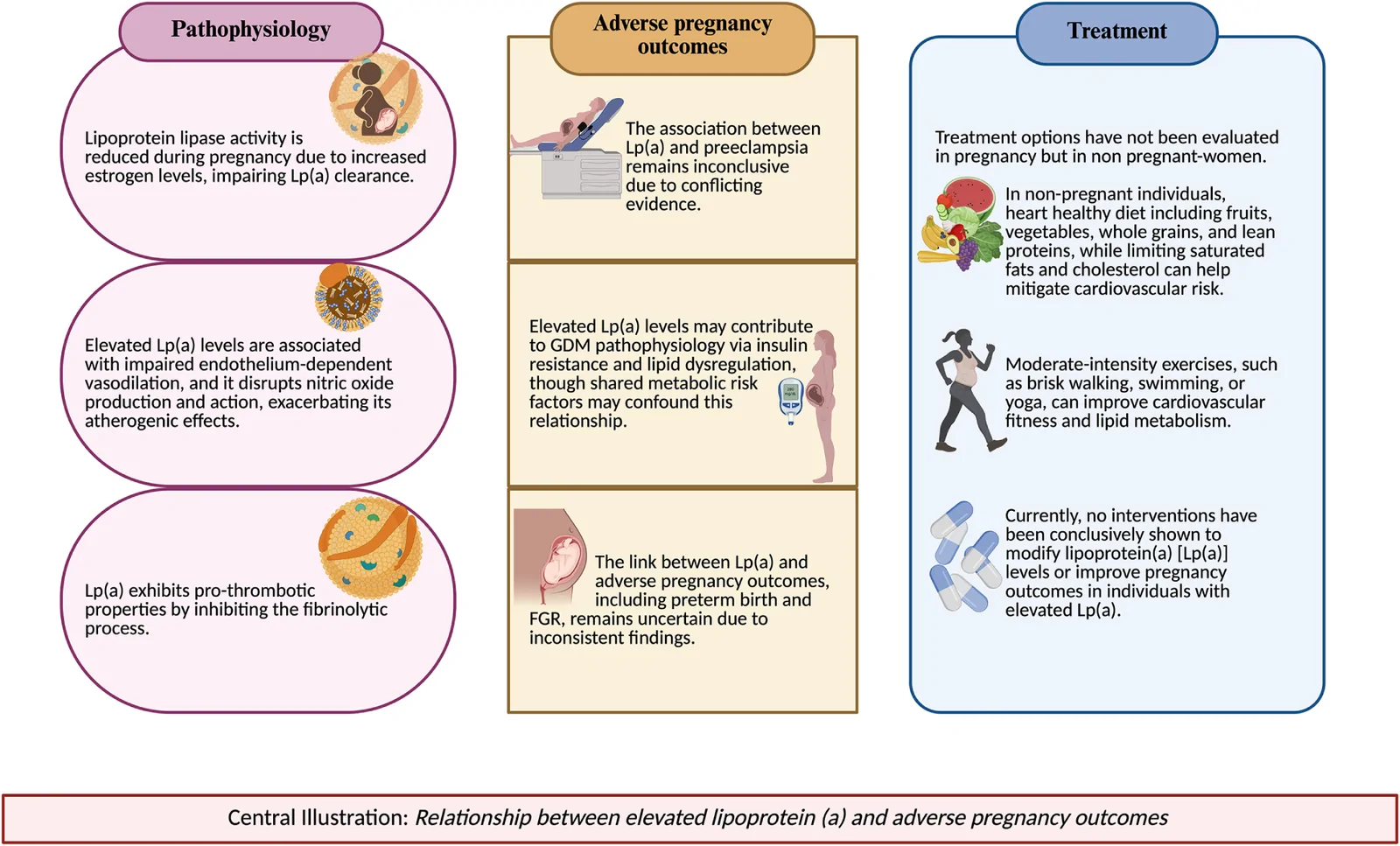
Central illustration.

Relationship between elevated lipoprotein(a) and adverse pregnancy outcomes. Created with https://biorender.com/. Ugoala, O. (2025).

**Table 1:** Summary of Lp(a) association with adverse pregnancy outcomes: evidence base, proposed mechanisms, and evidence strength.

Adverse pregnancy outcome	Key studies/evidence base	Proposed mechanism(s)	Evidence strength
HDP/PE	Konrad *et al*[28]: Lp(a) ≥ 40.5 mg/dL predicted progression to severe PE. Baksu *et al*[29]: no significant difference vs normotensive controls. Sattar *et al*[30]: no notable difference at 32 weeks.	Endothelial dysfunction, impaired NO bioavailability, pro-thrombotic effects on uteroplacental vasculature.	Weak/inconclusive
GDM	Wang *et al*[32]: elevated Lp-PLA2 in GDM (indirect marker; not direct Lp(a) measurement). Direct Lp(a) measurement studies are limited and inconsistent.	Insulin resistance increases lipoprotein synthesis; IL-6-mediated upregulation of hepatic Lp(a). (Biologically plausible but not yet clinically validated.)	Weak/inconclusive
FGR	Bar *et al*[33] and nested case-control (*n* = 82): elevated Lp(a) in FGR. Manten *et al* (2007): no significant differences. Retrospective data: Lp(a) up to 2.5 × higher in women with prior stillbirth.	Hypofibrinolytic and pro-thrombotic effects may compromise uteroplacental circulation, impairing fetal nutrient delivery. (Proposed mechanism.)	Moderate (limited by study heterogeneity)
Preterm birth	Ogunyemi et al[34], Mori *et al*[35]: elevated Lp(a) in women with preterm birth. Manten *et al*[36]: inconsistent. Many studies aggregate outcomes, limiting specificity.	Pro-thrombotic state leading to uteroplacental insufficiency; possible inflammatory mediation via Lp(a)-driven endothelial activation. (Proposed mechanism.)	Weak/inconclusive

HDP, hypertensive disorders of pregnancy; GDM, gestational diabetes mellitus; FGR, fetal growth restriction; PE, preeclampsia; NO, nitric oxide; IL-6, interleukin-6.

### Lp(a) and gestational diabetes

A cross-sectional analysis by Wang *et al* found that lipoprotein-associated phospholipase A2 (Lp-PLA2), an enzyme predominantly associated with Lp(a) and LDL, was significantly elevated in people with gestational diabetes mellitus (GDM) compared to people without GDM[32], suggesting a potential association with metabolic dysregulation in GDM. However, an important limitation of this study is that Lp-PLA2 is associated with Lp(a) and LDL broadly and is not a direct measure of Lp(a) concentration. These findings, therefore, cannot be taken as direct evidence of elevated Lp(a) in GDM. Direct measurement studies of Lp(a) in GDM populations are limited, and the available evidence does not establish a consistent, independent association[32].

The association between Lp(a) and GDM appears to be complex and potentially bidirectional. While GDM may be associated with elevated Lp(a) levels, pre-existing high Lp(a) concentrations might predispose people to GDM. The underlying mechanisms, though not fully elucidated, may involve insulin resistance, inflammation, and altered lipid metabolism. Insulin resistance, a hallmark of GDM, can disrupt lipid metabolism and increase the synthesis of lipoproteins, including Lp(a)[37].

Furthermore, the implications of elevated Lp(a) levels in GDM extend beyond pregnancy. People with a history of GDM are at increased risk of developing type 2 diabetes mellitus and cardiovascular disease later in life. This increased risk underscores the importance of understanding the role of Lp(a) in GDM for both immediate and long-term maternal health outcomes[32]. In summary, the evidence for a direct, independent role of Lp(a) in GDM pathophysiology remains limited and inconsistent. The observed associations are likely confounded by shared metabolic risk factors between GDM and dyslipidemia, and available studies have not consistently used direct Lp(a) measurements. Robust, prospective studies with direct Lp(a) quantification and appropriate adjustment for BMI and insulin resistance are needed before firm conclusions can be drawn (see Table 1 and Figure 1).

### Association between elevated Lp(a), PE, and GDM in maternal obesity

In the context of maternal obesity, the relationship between elevated Lp(a) levels and adverse pregnancy outcomes such as PE and GDM remains incompletely understood. While elevated Lp(a) has been associated with increased PE severity, as shown by Konrad *et al*, who reported that levels >40.5 mg/dL predicted progression from mild to severe disease, these findings excluded women with BMI ≥30 kg/m^2^, limiting their relevance to obese populations[28]. Furthermore, most studies investigating Lp(a) in pregnancy do not stratify by BMI, hindering efforts to determine whether obesity modifies Lp(a)-related risk. Although Lp(a) levels rise physiologically throughout gestation, peaking in the third trimester, it remains unclear whether this trajectory is amplified in the setting of obesity. In GDM, the role of Lp(a) is similarly uncertain, with limited and inconsistent data confounded by the metabolic disturbances intrinsic to obesity.

The following mechanistic pathways are biologically plausible and supported by indirect evidence in non-pregnant populations; however, their direct clinical relevance in pregnancy has not been prospectively validated. The mechanistic interplay between maternal obesity and Lp(a) involves multiple overlapping pathways. Obesity-associated chronic low-grade inflammation is characterized by elevated circulating interleukin-6 (IL-6), a cytokine known to upregulate hepatic Lp(a) and apolipoprotein(a) mRNA expression[24]. Upregulation of hepatic Lp(a) has been associated with endothelial dysfunction through increased oxidative stress and impaired nitric oxide bioavailability, mechanisms that may amplify Lp(a)-mediated vascular injury^[^17,19,20^]^. Visceral adiposity further drives insulin resistance, which exacerbates hepatic lipoprotein production and may amplify the physiological gestational rise in Lp(a)[2]. These convergent pathways suggest that obese pregnant women with elevated Lp(a) may face synergistic vascular dysfunction, potentially increasing susceptibility to PE and FGR beyond what either factor confers independently. The near-universal absence of BMI stratification in existing Lp(a)-pregnancy studies represents a critical methodological gap that future research must address.

### Lp(a) and FGR and preterm birth

The mechanisms by which Lp(a) contributes to preterm birth and FGR remain inadequately understood. It has been proposed – as a biologically plausible but not yet clinically validated mechanism – that Lp(a)’s hypofibrinolytic and prothrombotic properties may compromise uteroplacental circulation, potentially resulting in impaired fetal growth^[^2,34,35^]^. However, research on the association between Lp(a) and both spontaneous preterm birth and FGR has yielded mixed results. Some studies have identified elevated Lp(a) levels in pregnant women experiencing preterm birth, FGR, stillbirths, and other adverse pregnancy outcomes compared to those without such issues^[^33,34,36^]^. Conversely, other investigations found no significant differences in Lp(a) levels between women with FGR and those without[38]. A nested case-control study involving 82 pregnant women at risk for adverse pregnancy outcomes revealed significantly higher plasma Lp(a) concentrations in patients with preterm birth and FGR compared to those without these outcomes[33]. Furthermore, a retrospective analysis indicated that Lp(a) levels were up to 2.5 times greater in women with a history of stillbirth[39]. A notable limitation in many of these studies is the tendency to aggregate various adverse pregnancy outcomes, complicating the understanding of Lp(a)’s specific role in each complication ^[^33,34^]^ (Table 1; Figure 1).

## Diagnostic challenges and prognostic implications

### Screening and assessment of Lp(a) levels in pregnancy

Due to the recognized risks linked to elevated Lp(a) levels, the recent ACC/AHA guidelines recommend universal, one-time screening for Lp(a) in all adults for ASCVD risk assessment (class I recommendation)[40]. Additionally, some experts have stated that measuring Lp(a) could be beneficial for non-pregnant women with a family history of cardiovascular disease or previous pregnancy complications[41]. Lp(a) testing is most appropriately considered in women with: personal or family history of premature CVD or elevated Lp(a); prior adverse pregnancy outcomes (PE, FGR, and recurrent pregnancy loss); and additional cardiovascular risk factors (familial hypercholesterolemia, maternal obesity, and pre-existing dyslipidemia)^[^1,41,42^]^. Early detection of elevated Lp(a) levels in non-pregnant women with a family history of cardiovascular disease may enable closer monitoring and targeted interventions to reduce long-term cardiovascular risks. Screening in the first trimester has also been suggested to establish a baseline for tracking changes throughout pregnancy and assessing risks for conditions like PE, and even though there are no specific actionable points for elevated Lp(a), knowledge of this information can help pregnant women improve their overall health through lifestyle changes[1]. Although the European Society of Cardiology does not recommend routine screening for Lp(a) during pregnancy, recent guidelines from Poland advocate for Lp(a) measurement in all pregnant women, categorizing it as a class IIb/Level C recommendation aimed at identifying those at higher risk for pregnancy complications (e.g., PE, FGR, and recurrent pregnancy loss) and future cardiovascular events^[^1,42^]^. The National Lipid Association also emphasized that Lp(a) screening might be reasonable in pregnant women without prior testing, as elevated Lp(a) levels may impact overall management[43].

However, evaluating Lp(a) levels should be contextualized with other risk factors and clinical indicators. Integrating Lp(a) assessments with other emerging PE risk markers, such as uterine artery Doppler studies and serum biomarkers, may enhance predictive accuracy and redefine risk stratification, although further research is needed[44]. In the current absence of pregnancy-specific thresholds or validated interventions, elevated Lp(a) should not, in isolation, alter standard obstetric management but should inform a more comprehensive cardiovascular risk assessment. Women with elevated Lp(a) who are already recommended to take low-dose aspirin for PE prevention may warrant particular attention regarding timing of initiation and monitoring intensity^[^45–49^]^. The most actionable window for Lp(a)-directed management remains the preconception period. While aspirin remains the primary preventive strategy, Lp(a) measurement could help identify a subset of high-risk individuals who may benefit from more intensive monitoring or future targeted interventions as additional therapies emerge.

Distinguishing the expected physiological rise in Lp(a) during pregnancy from a clinically significant elevation remains an unresolved challenge. In non-pregnant individuals, the European Atherosclerosis Society defines a threshold of ≥50 mg/dL (≥125 nmol/L) as conferring high cardiovascular risk[8]. However, applying this static threshold during pregnancy is problematic, given that Lp(a) nearly doubles physiologically between weeks 10 and 35. The study by Konrad *et al* identified ≥40.5 mg/dL as predictive of progression from non-severe to severe PE[28], though that cohort excluded women with BMI ≥30 kg/m^2^, limiting generalizability. No validated, trimester-specific, pregnancy-adjusted threshold currently exists. Establishing one through large, prospective, BMI-stratified studies is a high-priority research objective and a prerequisite for integrating Lp(a) meaningfully into clinical pregnancy risk algorithms.

Proposed conceptual framework for Lp(a) interpretation in pregnancy:

Tier 1 – Establish Baseline: A pre-pregnancy or first-trimester Lp(a) measurement (ideally before 10 weeks’ gestation) documents the constitutional level before the physiological rise. In non-pregnant individuals, ≥50 mg/dL (≥125 nmol/L) is defined as high-risk by the European Atherosclerosis Society[8].

Tier 2 – Account for Physiological Rise: Lp(a) is expected to rise substantially during gestation, nearly doubling between weeks 10 and 35^[^1,2,10^]^. Serial measurements consistent with this trajectory, in the absence of other risk factors, should not prompt alarm in isolation.

Tier 3 – “Red Flag” Scenarios Warranting Heightened Vigilance: (a) Very high baseline Lp(a) (≥50–75 mg/dL) with additional cardiovascular comorbidities (familial hypercholesterolemia, hypertension, maternal obesity, and prior adverse pregnancy outcome); (b) Lp(a) ≥ 40.5 mg/dL in the context of established non-severe PE[28], acknowledging this threshold excludes BMI ≥30 kg/m^2^ and requires replication; (c) markedly elevated Lp(a) combined with impaired uterine artery Doppler velocimetry[44].

## Therapeutic interventions and management strategies

### Multidisciplinary approach to care

Preconception or antenatal counseling plays a vital role within a multidisciplinary strategy for managing elevated Lp(a) levels and minimizing adverse pregnancy outcomes. By fostering collaborative discussions among obstetricians, cardiologists, and genetic counselors, healthcare professionals enable individuals to make well-informed reproductive health choices.

### Lifestyle modifications

Managing elevated Lp(a) levels requires a multifaceted approach, with dietary adjustments playing a critical role. While direct evidence in pregnant populations is limited, studies in non-pregnant individuals suggest that a heart-healthy diet that includes fruits, vegetables, whole grains, and lean proteins while limiting saturated fats and cholesterol can help mitigate cardiovascular risk^[^32,37^]^. Although it was previously believed that dietary changes did not affect Lp(a) levels, emerging evidence suggests otherwise[50]. Research in non-pregnant individuals indicates that a reduction in saturated fatty acids (SFAs) can lead to elevated Lp(a) levels, while a slight increase in plant-derived SFAs may decrease Lp(a)[51]. Additionally, incorporating antioxidant-rich foods and omega-3 fatty acids can support cardiovascular health and modulate Lp(a) levels[52]. While the applicability of these findings in pregnancy remains uncertain, encouraging pregnant women to adopt these dietary modifications may contribute to overall health^[^53–55^]^.

While no studies have directly evaluated whether low-carbohydrate diets can regulate Lp(a) concentrations or inflammatory markers in women with GDM, emerging evidence suggests that carbohydrate restriction may confer metabolic benefits. In particular, a recent trial demonstrated that carbohydrate restriction improved insulin-resistant dyslipoproteinemia and lowered Lp(a) levels without adversely affecting LDL cholesterol or inflammation, with results remaining significant even after conservative adjustment for multiple comparisons[50]. Low-carbohydrate diets are already widely used in the management of GDM, where they have been shown to improve glycemic control and reduce postprandial glucose excursions^[^56,57^]^. However, whether these dietary interventions also modulate Lp(a) or inflammation in this population remains unknown. These knowledge gaps underscore the need for BMI-stratified, longitudinal studies to elucidate the interaction between Lp(a), obesity, dietary patterns, and adverse pregnancy outcomes.

In addition to dietary management, regular physical activity supports overall cardiovascular health in pregnant women with elevated Lp(a). Moderate-intensity exercises, such as brisk walking, swimming, or yoga, can improve cardiovascular fitness and lipid metabolism[58]. However, while exercise is generally beneficial for maternal well-being, evidence supporting its role in preventing adverse pregnancy outcomes remains inconclusive, with most intervention studies showing limited success. Additionally, although evidence remains inconclusive, smoking cessation might be beneficial in managing elevated Lp(a) levels, as smoking is associated with increased cardiovascular risks and APOs, including PE and preterm birth^[^59–62^]^.

### Pharmacological interventions

Statins, niacin, and proprotein convertase subtilisin/kexin type 9 (PCSK9) inhibitors have shown effectiveness in reducing Lp(a) levels and enhancing lipid profiles in non-pregnant populations^[^63,64^]^. Nonetheless, the limited safety data and potential teratogenic effects associated with these agents during pregnancy preclude their recommendation as a first-line treatment^[^64,65^]^. Hence, their use during pregnancy is not recommended. Statins are generally contraindicated in pregnancy due to the theoretical risk of disrupting fetal cholesterol biosynthesis, which is essential for normal neural and adrenal development[65]; their use should be discontinued prior to conception or as soon as pregnancy is confirmed. PCSK9 inhibitors, while promising as Lp(a)-lowering agents in the general population, lack adequate safety and efficacy data in pregnancy; available case reports describe inadvertent first-trimester exposure without confirmed teratogenicity[64], but this evidence base is far too limited to support their use during gestation. In light of these constraints, the most clinically actionable window for pharmacological optimization remains the preconception period. Women with markedly elevated Lp(a) and planned pregnancy should be identified and referred to a multidisciplinary team involving cardiology, maternal-fetal medicine, and endocrinology prior to conception, where evidence-based cardiovascular risk reduction can be implemented without the safety limitations imposed by pregnancy itself[66]. In addition to lipid-lowering medications, aspirin use has been identified as a potential pharmacological option for managing elevated Lp(a) levels in non-pregnant individuals in a prospective cohort study, although randomized controlled studies are needed[67]. Commonly used to prevent PE, low-dose aspirin has demonstrated significant benefits in this context^[^45–49^]^. Although its direct effects on Lp(a) levels remain less established, its antiplatelet and anti-inflammatory properties may enhance cardiovascular health during pregnancy^[^68,69^]^. Since pregnant women at high risk for PE are already advised to take low-dose aspirin, those with elevated Lp(a) may warrant additional attention beyond routine prophylaxis. Given Lp(a)’s role in vascular dysfunction and long-term cardiovascular risk, this subgroup may benefit from earlier initiation of aspirin, closer monitoring for PE development, and a more comprehensive cardiovascular risk assessment during and after pregnancy. Future research should explore whether specific aspirin dosing strategies or additional interventions could further mitigate risk in individuals with elevated Lp(a).

### Future directions and research opportunities

Management of elevated Lp(a) primarily targets the reduction of LDL cholesterol levels, but traditional pharmacological strategies have demonstrated limited effectiveness and considerable adverse effects in the general population. As a result, innovative therapies are under investigation. Despite significant advancements in understanding disease mechanisms in non-pregnant populations, the study of treatment efficacy and disease progression during pregnancy remains severely understudied. Pregnant women are often excluded from clinical research due to ethical and safety concerns, yet this exclusion limits our ability to determine whether interventions truly improve maternal and fetal outcomes. Before novel therapies can be considered, fundamental questions remain unanswered, such as the impact of existing treatments on pregnancy-specific pathophysiology and whether modifying disease processes during pregnancy alters long-term outcomes. A critical step forward is to shift the paradigm from exclusion to inclusion, ensuring that pregnant women are actively represented in research to develop evidence-based strategies that optimize both maternal and neonatal health.

Among these, antisense oligonucleotides and small interfering RNAs (siRNAs) have emerged as promising agents that utilize gene silencing, achieving a reduction in plasma Lp(a) levels of over 90%^[^70–72^]^. However, these antisense oligonucleotides and siRNAs have not been studied in pregnant women due to safety and ethical considerations. Future studies on aspirin’s impact on Lp(a) levels in pregnant women may also be beneficial, particularly through randomized controlled trials[73].

Several limitations of this review warrant acknowledgment. First, as this is a narrative rather than a systematic review, no formal risk-of-bias assessment was conducted, and findings were not subjected to meta-analytic synthesis. The structured search strategy, while reported with quantitative detail for transparency, does not confer systematic review status, and the screening figures provided are approximations reflecting the scope of the search. Second, the primary literature is itself heterogeneous, with considerable variation in sample size, gestational timing of Lp(a) measurement, assay methodology, outcome definitions, and adjustment for confounders including BMI, renal function, and pre-existing dyslipidemia. Third, the near-universal exclusion of pregnant women from clinical trials limits the translational applicability of both mechanistic insights and therapeutic data derived from non-pregnant populations. These limitations collectively underscore the need for adequately powered, prospective, pregnancy-specific studies before definitive clinical conclusions can be drawn.

In conclusion, this review highlights that, despite the well-established cardiovascular risk conferred by elevated Lp(a), its role in adverse pregnancy outcomes remains incompletely characterized. The evidence linking Lp(a) to HDP and gestational diabetes is inconsistent, reflecting genuine methodological heterogeneity across studies rather than a settled biological question. The physiological rise in Lp(a) during gestation complicates risk stratification, and no validated pregnancy-specific threshold currently exists. Current pharmacological options are limited by safety concerns in pregnancy, while lifestyle interventions offer modest and indirect benefit. Novel Lp(a)-lowering therapies, including antisense oligonucleotides and siRNA agents, show substantial promise, but have not been studied in pregnant populations. Addressing this evidence gap requires adequately powered, prospective studies that include pregnant women, incorporate direct Lp(a) measurement, stratify by BMI, and distinguish between individual adverse pregnancy outcomes rather than aggregating them. Until such evidence is available, clinical decision-making regarding Lp(a) in pregnancy must be guided by individual risk assessment within a multidisciplinary framework.

## Data Availability

Not applicable.
